# Destin2: Integrative and cross-modality analysis of single-cell chromatin accessibility data

**DOI:** 10.3389/fgene.2023.1089936

**Published:** 2023-02-17

**Authors:** Peter Y. Guan, Jin Seok Lee, Lihao Wang, Kevin Z. Lin, Wenwen Mei, Li Chen, Yuchao Jiang

**Affiliations:** ^1^ Department of Biostatistics, University of North Carolina at Chapel Hill, Chapel Hill, NC, Unites States; ^2^ Department of Genetics, University of North Carolina at Chapel Hill, Chapel Hill, NC, Unites States; ^3^ Department of Statistics, University of Pennsylvania, Philadelphia, PA, Unites States; ^4^ Department of Biostatistics, University of Florida, Gainesville, FL, Unites States; ^5^ Lineberger Comprehensive Cancer Center, University of North Carolina at Chapel Hill, Chapel Hill, NC, Unites States

**Keywords:** single-cell epigenomics, single-cell ATAC-seq, chromatin accessibility, single-cell multiome, cross-modality integration

## Abstract

We propose Destin2, a novel statistical and computational method for cross-modality dimension reduction, clustering, and trajectory reconstruction for single-cell ATAC-seq data. The framework integrates cellular-level epigenomic profiles from peak accessibility, motif deviation score, and pseudo-gene activity and learns a shared manifold using the multimodal input, followed by clustering and/or trajectory inference. We apply Destin2 to real scATAC-seq datasets with both discretized cell types and transient cell states and carry out benchmarking studies against existing methods based on unimodal analyses. Using cell-type labels transferred with high confidence from unmatched single-cell RNA sequencing data, we adopt four performance assessment metrics and demonstrate how Destin2 corroborates and improves upon existing methods. Using single-cell RNA and ATAC multiomic data, we further exemplify how Destin2’s cross-modality integrative analyses preserve true cell-cell similarities using the matched cell pairs as ground truths. Destin2 is compiled as a freely available R package available at https://github.com/yuchaojiang/Destin2.

## Introduction

Recent advances in single-cell assay of transposase-accessible chromatin followed by sequencing (scATAC-seq) technologies (Buenrostro et al., 2015; Cusanovich et al., 2015; Satpathy et al., 2019) offer unprecedented opportunities to characterize cellular-level chromatin accessibilities and have been successfully applied to atlas-scale datasets to yield novel insights on epigenomic heterogeneity (Cusanovich et al., 2018; Domcke et al., 2020). scATAC-seq data analysis presents unique methodological challenges due to its high noise, sparsity, and dimensionality (Urrutia et al., 2019). Multiple statistical and computational methods have been developed and evaluated by independent benchmark studies (Chen et al., 2019).

The first set of methods call ATAC peaks or segment the genome into bins and take the cell by peak (or cell by bin) matrix as input. Among these methods, Signac (Stuart et al., 2021), scOpen (Li et al., 2021), and RA3 (Chen et al., 2021) perform TF-IDF normalization followed by different dimension reduction techniques. SnapATAC (Fang et al., 2021) computes a Jaccard similarity matrix, while cisTopic (Bravo Gonzalez-Blas et al., 2019) performs topic modeling. Moving beyond the peak matrix, Cicero (Pliner et al., 2018) and MAESTRO (Wang et al., 2020) make gene expression predictions from unweighted and weighted sum of the ATAC reads in gene bodies and promoter regions, respectively; the predicted gene activities have been shown to in the ballpark recapitulate the transcriptomic profiles and discern cell populations (Jiang et al., 2022). For TF-binding motifs, chromVAR (Schep et al., 2017) computes a motif deviation score by estimating the gain or loss of accessibility within peaks sharing the same motif relative to the average cell profile; these deviation scores have also been shown to enable accurate clustering of scATAC-seq data.

Notably, most, if not all, of the aforementioned methods carry out “unimodal” analysis with a single type of feature input (i.e., peaks, genes, or motifs). One of the earliest methods, SCRAT (Ji et al., 2017), proposes to use empirical and prior knowledge to aggregate the peaks into genes, motifs, and gene sets, while neglecting the peak-level information due to high computational burden. EpiScanpy (Danese et al., 2021), ArchR (Granja et al., 2021), and Signac (Stuart et al., 2021) all generate multimodal feature inputs. However, dimension reduction and clustering are still focused on the peak accessibilities—the gene activities are generally integrated with single-cell RNA sequencing (scRNA-seq) data for alignment, and the motif deviation scores are used to identify enriched and/or differentially accessible motifs.

To our best knowledge, no integrative methods are available for a cross-modality analysis of scATAC-seq data, yet it has been shown that the peaks, genes, and motifs all contain signals to separate the different cell types/states. Here, we propose Destin2, a successor to our previous unimodal method Destin (Urrutia et al., 2019), for cross-modality dimension reduction, clustering, and trajectory reconstruction for scATAC-seq data. The framework integrates cellular-level epigenomic profiles from peak accessibility, motif deviation score, and pseudo-gene activity and learns a shared manifold using the multimodal input. We apply the method to real datasets with both discretized cell types and transient cell states and carry out benchmarking studies to demonstrate how Destin2’s cross-modality integration corroborates and improves upon existing methods based on unimodal analyses.

## Materials and methods

**FIGURE 1:**
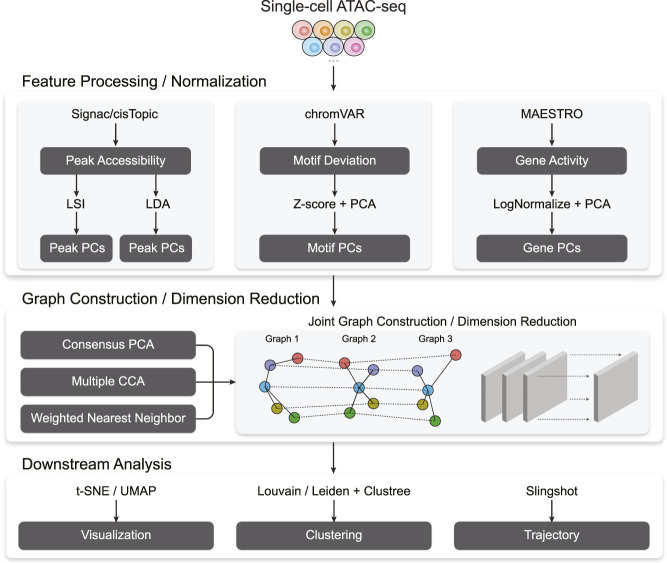
A flowchart outlining the procedures for cross-modality scATAC-seq analysis by Destin2.

Figure 1 outlines Destin2’s analytical framework. For unimodal data input, Destin2 utilizes Signac (Stuart et al., 2021), MAESTRO (Wang et al., 2020), and chromVAR (Schep et al., 2017) for pre-processing and generating the matrices of peak accessibility, gene activity, and motif deviation, where the cell dimensions are matched, and yet the feature dimensions differ. The peak matrix can be directly loaded from the output of cellranger-atac or called/refined by MACS2 (Zhang et al., 2008). Pseudo-gene activities can be derived from either taking the sum of ATAC reads in gene bodies and promoter regions by Signac (Stuart et al., 2021) or using a regulatory potential model that sums ATAC reads weighted based on existing gene annotations by MAESTRO (Wang et al., 2020). Motif deviation scores are computed using chromVAR (Schep et al., 2017) and measure the deviation in chromatin accessibility across the set of peaks containing the TF-binding motifs, compared to a set of background peaks. Destin2, by its default, uses the JASPAR database for pairs of TF and motif annotation in vertebrates (Fornes et al., 2020).

For data normalization and dimension reduction, we adopt two parallel and state-of-the-art approaches, latent semantic indexing (LSI) and latent Dirichlet allocation (LDA), for the peak matrix. LSI normalizes reads within peaks using the term frequency-inverse document frequency transformation (TF-IDF), followed by a PCA-based dimension reduction (Stuart et al., 2021). LDA is a topic modeling approach commonly used in natural language processing and has been successfully applied to scATAC-seq data to identify cell states from topic-cell distribution and explore *cis*-regulatory regions from region-topic distribution by cisTopic (Bravo Gonzalez-Blas et al., 2019). For the motif and gene matrix, we use z -score transformation and the LogNormalize function by Seurat (Butler et al., 2018), followed by principal component analysis (PCA), respectively. These within-modality normalization and dimension reduction, which return peak principal components (PCs), motif PCs, and gene PCs, are necessary. They effectively reduce signal-to-noise ratios, and more importantly, it has been shown that PCA, followed by canonical correlation analysis (CCA), offers a powerful approach to uncover latent structure shared across modalities through an integrative analysis (Brown et al., 2018). The number of PCs can be chosen by inspecting the variance reduction (i.e., elbow) plot or using the JackStraw method (Satija et al., 2015), which randomly permutes a subset of the data and compares the PCs for the permuted data with the observed PCs to determine statistical significance.

With the pre-processed and normalized unimodal data input, Destin2 offers three options for cross-modality integration: consensus PCA (CPCA), generalized/multiple CCA (MultiCCA), and weighted nearest neighbor (WNN). Denote the feature input across K modalities as $X^{\left(1\right)}\in R^{n\times p_{1}},\ldots ,X^{\left(K\right)}\in R^{n\times p_{K}}$ , where the n cells are matched. (I) CPCA (Westerhuis et al., 1998), algebraically equivalent to applying a second-step PCA to the concatenated peak PCs, motif PCs, and gene PCs, returns consensus PCs as joint dimension reductions, which reveal the union of the latent structure across multiple modalities. To identify the first-rank consensus PC is analogous to solve:

$$\left\{{\hat{w}}^{\left(1\right)},\ldots ,{\hat{w}}^{\left(K\right)}\right\}=\mathrm{argmin}{\left\|\left[X^{\left(1\right)},\ldots ,X^{\left(K\right)}\right]-\left[X^{\left(1\right)},\ldots ,X^{\left(K\right)}\right]\left[w^{\left(1\right)},\ldots ,w^{\left(K\right)}\right]{\left[w^{\left(1\right)},\ldots ,w^{\left(K\right)}\right]}^{T}\right\|}^{2},$$

such that $w^{\left(k\right)}\in R^{p_{k}}$ for 1≤k≤K and $\left\|w^{\left(1\right)}\right\|+\ldots +\left\|w^{\left(K\right)}\right\|=1.$ (II) MultiCCA (Kettenring, 1971), on the other hand, finds maximally correlated linear combinations of the features between each pair of modalities by solving:

$$\left\{{\hat{w}}^{\left(1\right)},\ldots ,{\hat{w}}^{\left(K\right)}\right\}=\mathrm{argmax}\sum_{1\leq i<j\leq K}{w^{\left(i\right)}}^{T}{X^{\left(i\right)}}^{T}X^{\left(j\right)}w^{\left(j\right)},$$

such that $w^{\left(k\right)}\in R^{p_{k}}$ and ${w^{\left(k\right)}}^{T}{X^{\left(k\right)}}^{T}X^{\left(k\right)}w^{\left(k\right)}=1$ for 1≤k≤K ; we utilize the implementation from the mogsa package (Meng et al., 2019) in R. (III) Instead of optimizing for the modality- and feature-specific loading vector for projections of the three modalities, the recently developed WNN method (Hao et al., 2021) learns cell- and modality-specific weights, which reflect the information content for each modality and are used to calculate a weighted cell-cell similarity measure and construct a WNN graph. We will not go into the algorithmic details of the WNN method—readers can refer to the Seurat V4 publication (Hao et al., 2021), where the WNN framework is extended to more than two modalities with matched cells.

Followed by joint dimension reduction and graph construction, tSNE/UMAP can be used for visualization. Destin2 adopts Louvain/Leiden clustering (Traag et al., 2019) for community detection and identification of discrete cell clusters. The number of cell clusters (i.e., the resolution parameter) can be optimized using the clustree method (Zappia and Oshlack, 2018), which builds a tree to visualize and examine how clusters are related to each other at varying resolutions, allowing researchers to assess which clusters are distinct and which are unstable with the use of additional metrics such as the SC3 stability index (Kiselev et al., 2017). For cell population exhibiting continuous and connected cell states, Destin2 resorts to a flexible and modularized approach, Slingshot (Street et al., 2018), for trajectory reconstruction; smooth representation of the lineages and pseudotime values are inferred using the joint dimension reduction and visualized on the UMAP space.

## Results

### Destin2 improves clustering accuracy compared to unimodal analysis methods

We apply Destin2 to four scATAC-seq datasets of human peripheral blood mononuclear cells (PBMCs) from 10x Genomics, adult mouse cortex cells from 10x Genomics, human bone marrow mononuclear cells (BMMCs) (Granja et al., 2019), and human fetal organs (Domcke et al., 2020). See Supplementary Table S1 for summary and details of the data. For the PBMC and adult mouse cortex datasets, we annotate cell types using scRNA-seq experiments from the same biological systems (PBMC from 10x Genomics and mouse brain from the Allen Brain Institute), utilizing the CCA-based method for cross-modality integration and label transfer (Stuart et al., 2019) and only keeping cells that can be uniquely and confidently assigned to one cell type. For the BMMC dataset, we use the curated cell type labels from the original publication (Granja et al., 2019). For the human fetal dataset, we resort to the tissues of origin from the experimental design/sample collection. These cell types/tissues are used as ground truths for performance assessment.

We apply unimodal analysis methods (i.e., peak analysis by Signac and cisTopic, motif analysis by chromVAR, and gene activity analysis by Signac/MAESTRO) and Destin2 to these datasets, with UMAP visualizations shown in Supplementary Figure S1. For benchmarking, we adopt four metrics for performance assessment. (I) Adjusted rand index (ARI) is used to compare the identified cell clusters against the annotated cell types, with 1 indicating that the two are exactly the same. (II) Adjusted mutual information (AMI) is similar to ARI but is more suited when there exist small and unbalanced clusters (Romano et al., 2016). (III) Homogeneity score (H-score) is an entropy-based measure of the similarity between two clusterings and ranges between 0 and 1, where 1 indicates perfect homogeneity. (IV) Cell-type local inverse Simpson’s index (cLISI) (Korsunsky et al., 2019) is used to assess the degree of mixing/separation of annotated cell types, with 1 indicating that the different cell types group separately and 2 indicating that the different cell types erroneously group together.

**FIGURE 2:**
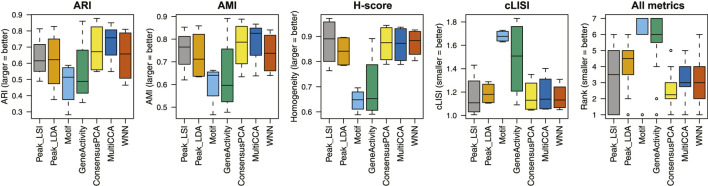
Benchmarking clustering accuracy. Four different metrics—ARI, AMI, H-score, and cLISI—were used for performance assessment. For each metric and each method, results from the four unimodal scATAC-seq datasets are aggregated (i.e., each boxplot contains four data points). The ranks of the methods for each metric are computed and then combined across all metrics and datasets. Destin2’s multimodal analysis framework achieves the highest rank and improves clustering accuracy compared to conventional unimodal analysis methods.

Across the four scATAC-seq datasets, our results suggest that the multimodal analysis methods proposed by Destin2 improve clustering accuracy compared to conventional unimodal analysis methods using ARI and AMI as assessment metrics (Figure 2; Supplementary Table S2). For cLISI and H-score, since the gold-standard cell-type labels are transferred using the LSI-based dimension reduction as weights, it is not surprising that the LSI method achieves the top performance; non-etheless, the difference between LSI-based methods and Destin2’s cross-modality integration results are negligible (Figure 2; Supplementary Table S2). Note that while the motif analysis returns the seemingly worst result, whether the motif modality is included in the integrative analysis does not significantly alter the output (Supplementary Figure S2), demonstrating Destin2’s robustness to the differential information content across modalities. Additionally, and more importantly, careful inspection of the confusion matrix (shown as a heatmap in Supplementary Figure S3) suggests that Destin2 is able to identify cell types/states that are otherwise indistinguishable and/or wrongly classified from a unimodal analysis—e.g., Lamp5 v.s. Vip in Supplementary Figure S3B, as well as GMP v.s. CD14 monocytes and CLP v.s. pre-B cells in Supplementary Figure S3C.

For downstream analysis, we first demonstrate how to determine the clustering resolution using the clustree method (Supplementary Figure S4). Specifically, clustering results with varying clustering resolutions [and thus varying SC3 stability measures (Kiselev et al., 2017)] are visualized as a tree: new clusters form from existing clusters, and the overlap in cells between clusters at adjacent resolutions is computed and used to calculate the in-proportion for each edge. Unstable clusters result in cells switching between branches of the trees, with low in-proportion edges; one can thus infer which areas of the tree are likely to be the result of true clusters and which are caused by over-clustering (Zappia and Oshlack, 2018). For cell populations with continuous cell states, we further demonstrate how to reconstruct the development/differentiation trajectory using Destin2’s joint dimension reduction paired with the Slingshot method. As an example, we show the reconstruction of the true branching lineages during human hematopoietic differentiation using the BMMC data (Supplementary Figure S5).

### Destin2 better preserves cell-cell similarities using single-cell RNA and ATAC multiomic data

We further applied Destin2 to three single-cell RNA and ATAC multiomic datasets of human PBMCs from 10x Genomics, adult mouse cortex cells from 10x Genomics, and mouse skin data from SHARE-seq (Ma et al., 2020). See Supplementary Table S1 for a data summary. In using these multiomic datasets, we demonstrate how Destin2’s cross-modality analyses preserve true cell-cell similarities by using the matched cell information as ground truth. Importantly, this also does not need the RNA-ATAC alignment or the transfer of discretized cell-type labels, which often fails for cell populations consisting of transient states.

Specifically, we first obtained the low-dimensional embedding using the ATAC data alone by the various unimodal methods and Destin2’s multimodal methods. We then reconstructed the low-dimensional embedding using the RNA data using Seurat’s scRNA-seq processing pipeline (Butler et al., 2018). We then assess how the low-dimensional embedding by the ATAC domain agrees with that by the RNA domain. For benchmarking, we adopt two additional metrics designed specifically for the single-cell multiomic data—fraction of samples closer than the nearest neighbor (FOSCTTNN) and agreement. Both metrics measure the preservation of a cell’s nearest neighbors between the RNA and ATAC domains and do not rely on annotated cell types or identified clusters. FOSCTTNN is adapted from the “fraction of samples closer than the true match” metric (Liu et al., 2019): for each cell, we first identify its nearest neighbor (i.e., closest cell) in the RNA domain as ground truth, and then, in the ATAC domain, we calculate the fraction of cells that are closer than its true nearest neighbor. For agreement (Welch et al., 2019), we identify each cell’s k nearest neighbors in the RNA and ATAC domains, respectively, and then calculate the fraction of overlap. Nearest neighbors are identified using Euclidean distance of the cells’ reduced dimension from each modality and method. The two cell-specific metrics can be further summarized using median and Gini mean difference (GMD) across cells.

For the three multiomic datasets, our results suggest that the multimodal analysis methods offered by Destin2 exhibit top or near-top performance. For FOSCTTNN, Destin2’s cross-modality integration results are either top-performing or negligibly different from the top performer (Table 1); for agreement across different k (number of nearest neighbors), the WNN method achieves the top performance (Table 2). Interestingly and importantly, neither LSI nor LDA is indefinitely preferred from this benchmark analysis—e.g., LDA outperforms LSI using the FOSCTTNN metric in the PBMC data (Table 1), while LSI improves upon LDA using the agreement metric by a large margin in the mouse brain data (Table 2). In real data analysis, where there is no ground truth to guide method selection, Destin2 integrates and corroborates information from both methods and demonstrates its robustness.

**TABLE 1:** FOSCTTNN metrics on single-cell RNA and ATAC multiomic datasets. Destin2’s multimodal analyses achieve top or near-top performance. FOSCTTNN is bound between 0 and 1, with 0 being the best performance. Neither LSI nor LDA is indefinitely preferred from this benchmark analysis; Destin2 integrates and corroborates information across methods and modalities.

(A) PBMC 10x Genomics FOSCTTNN						
**Method**	**q1**	**Median**	**GMD**	**Mean**	**sd**	**q3**
Peak_LSI	0.015	0.045	0.107	0.089	0.126	0.116
Peak_LDA	0.013	0.039	0.081	0.069	0.095	0.090
Motif	0.048	0.131	0.168	0.174	0.158	0.261
GeneActivity	0.017	0.054	0.124	0.104	0.133	0.137
ConsensusPCA	0.014	0.045	0.098	0.084	0.107	0.118
MultiCCA	0.014	0.044	0.104	0.086	0.121	0.112
WNN	0.013	0.042	0.094	0.080	0.102	0.110
(B) Mouse Brain 10x Genomics FOSCTTNN						
**Method**	**q1**	**Median**	**GMD**	**Mean**	**sd**	**q3**
Peak_LSI	0.004	0.014	0.085	0.056	0.130	0.043
Peak_LDA	0.006	0.021	0.077	0.056	0.110	0.056
Motif	0.010	0.033	0.105	0.079	0.133	0.085
GeneActivity	0.028	0.109	0.308	0.257	0.294	0.487
ConsensusPCA	0.005	0.017	0.088	0.059	0.129	0.049
MultiCCA	0.005	0.017	0.083	0.057	0.122	0.050
WNN	0.005	0.018	0.091	0.062	0.130	0.055
(C) Mouse Skin SHARE-seq FOSCTTNN						
**Method**	**q1**	**Median**	**GMD**	**Mean**	**sd**	**q3**
Peak_LSI	0.011	0.034	0.165	0.117	0.196	0.110
Peak_LDA	0.011	0.036	0.143	0.104	0.174	0.101
Motif	0.024	0.076	0.185	0.154	0.194	0.200
GeneActivity	0.062	0.208	0.332	0.323	0.301	0.551
ConsensusPCA	0.011	0.034	0.147	0.105	0.180	0.097
MultiCCA	0.011	0.034	0.139	0.100	0.176	0.092
WNN	0.010	0.031	0.134	0.096	0.170	0.091

**TABLE 2:** Agreement metrics on single-cell RNA and ATAC multiomic datasets. Different numbers of nearest numbers ( k ) were selected. Destin2’s multimodal analyses, especially the WNN method, achieve top or near-top performance. Agreement is bound between 0 and 1, with 1 being the best performance. Neither LSI nor LDA is indefinitely preferred from this benchmark analysis; Destin2 integrates and corroborates information across methods and modalities.

(A) PBMC 10x Genomics Agreement							
**k**	**Peak_LSI**	**Peak_LDA**	**Motif**	**Gene Activity**	**Consensus PCA**	**MultiCCA**	**WNN**
50	0.091	0.102	0.032	0.085	0.092	0.091	0.107
100	0.163	0.175	0.061	0.150	0.163	0.163	0.183
150	0.220	0.232	0.086	0.200	0.219	0.221	0.241
200	0.269	0.282	0.110	0.244	0.268	0.270	0.290
(B) Mouse Brain 10x Genomics Agreement							
**k**	**Peak_LSI**	**Peak_LDA**	**Motif**	**Gene Activity**	**Consensus PCA**	**MultiCCA**	**WNN**
50	0.350	0.288	0.222	0.119	0.324	0.318	0.314
100	0.514	0.439	0.358	0.196	0.481	0.478	0.468
150	0.613	0.549	0.455	0.255	0.584	0.583	0.569
200	0.681	0.629	0.532	0.306	0.659	0.658	0.643
(C) Mouse Skin SHARE-seq Agreement							
**k**	**Peak_LSI**	**Peak_LDA**	**Motif**	**Gene Activity**	**Consensus PCA**	**MultiCCA**	**WNN**
50	0.046	0.048	0.024	0.010	0.046	0.046	0.050
100	0.088	0.088	0.047	0.020	0.087	0.085	0.094
150	0.124	0.122	0.068	0.030	0.123	0.122	0.131
200	0.157	0.154	0.087	0.039	0.156	0.155	0.166

## Discussion

We propose Destin2 to integrate multimodal peak accessibility, motif deviation, and pseudo-gene activity measures derived from scATAC-seq data. Destin2’s cross-modality integration can finish within a few minutes on a local computer; the computational bottleneck comes from the preprocessing step, which can also finish reasonably fast within an hour across tens of thousands of cells (Supplementary Table S3). For peak accessibility, Destin2 integrates two most popular techniques—LSI and LDA—for data pre-processing and within-modality dimension reduction. While Destin2 is not restricted to only taking peak accessibilities as input, our framework offers a strategy to ensemble results from the various peak-modeling methods (Chen et al., 2019), so long as method-specific dimension reductions are provided. For motif deviation, Destin2, by its default, resorts to chromVAR (Schep et al., 2017), which can be computationally intensive and infeasible to handle atlas-scale scATAC-seq data. While alternative methods are currently being developed for higher scalability, a viable shortcut solution is to process the entire data in mini batches (i.e., random subsamples of the cells), thus not requiring all the data to be loaded into memory at one time. Such a strategy has been successfully applied to scRNA-seq data with millions of cells (Hicks et al., 2021). For pseudo-gene activity, Destin2 aggregates ATAC reads over gene bodies and promoter regions using Signac (Stuart et al., 2021) or MAESTRO (Wang et al., 2020), yet this largely neglects peaks and fragments from intergenic and non-coding regions. Additional annotations, such as the enhancers (Shlyueva et al., 2014), super-enhancers (Pott and Lieb, 2015), A/B compartments (Lieberman-Aiden et al., 2009), and chromatin loops (Rao et al., 2014), can be easily incorporated into Destin2’s framework as additional modalities to be integrated.

For cross-modality joint modeling, Destin2 utilizes three statistically rigorous and computationally efficient methods—CPCA, MultiCCA, and WNN—and shows its outperformance and robustness. Additional methods that fall in the realm of multiomic integration [e.g., JIVE (Lock et al., 2013), MOFA (Argelaguet et al., 2018), *etc.*] have not been thoroughly explored. For CCA, its variants and extensions, such as sparse CCA (Witten et al., 2009) and decomposition-based CCA (Shu et al., 2020), can potentially further boost performance. Overall, we believe that the framework by Destin2 introduces the concept of multiomic integration to scATAC-seq data; through the various benchmarking studies, we exemplify its utility and benefit and illustrate how it can better facilitate downstream analyses.

## Data Availability

The datasets presented in this study can be found in online repositories. The names of the repository/repositories and accession number(s) can be found in the article/Supplementary Material.
